#  An efficient gene editing system for *Eustoma grandiflorum* based on somatic embryogenesis

**DOI:** 10.1093/hr/uhag197

**Published:** 2026-06-10

**Authors:** Chunlian Jin, Xingyu Zhou, Lingling Yuan, Fan Li, Jihua Wang

**Affiliations:** National Engineering Research Center for Ornamental Horticulture, Key Laboratory for Flower Breeding of Yunnan Province, Floriculture Research Institute, Yunnan Academy of Agricultural Sciences, Kunming 650205, China; Yunnan Seed Laboratory, Kunming 650205, China; School of Agriculture, Yunnan University, Kunming 650504, China; Yunnan Seed Laboratory, Kunming 650205, China; National Engineering Research Center for Ornamental Horticulture, Key Laboratory for Flower Breeding of Yunnan Province, Floriculture Research Institute, Yunnan Academy of Agricultural Sciences, Kunming 650205, China; Yunnan Seed Laboratory, Kunming 650205, China; National Engineering Research Center for Ornamental Horticulture, Key Laboratory for Flower Breeding of Yunnan Province, Floriculture Research Institute, Yunnan Academy of Agricultural Sciences, Kunming 650205, China; Yunnan Seed Laboratory, Kunming 650205, China

Dear editor,

*Eustoma grandiflorum* belonging to Gentianaceae, is one of the top 10 cut flowers cultivated mainly in America, China, Japan, and Netherlands, which is also called ‘prickle-less rose’. Compared with rose, the breeding history of *Eustoma* is quite short, resulting in modest cultivars released to date [1]. F1 hybridization is the primary breeding method for *Eustoma* whereas the transgenic breeding develops slowly. Traditional breeding methods are unable to precisely break through the bottlenecks in improving key traits, such as flower color, flower shape, floral scent, and stress resistance. In contrast, transgenic and gene editing technologies can precisely modify target genes and enable rapid creation of elite germplasm. As early as 1996, genetic transformation was conducted in leaf explants of *Eustoma* cultivar ‘SP3’ and ‘V3’ *via Agrobacterium tumefaciens* strain EHA105, but <10% of the explants were transformed (calculated by positive calli). Seven polymerase chain reaction (PCR)-positive plantlets were regenerated from this experiment and only one plantlet expressed the target protein [2]. This leaf disk transformation method was optimized by involving acetosyringone during *Agrobacterium* inoculation but the regeneration and transformation efficiency was still low [3]. To increase the transformation efficiency, flower dipping method was developed in *Eustoma* and the transformation efficiency reached 1.5%–3.7% as reported. Compared with the reported tissue culture-based transformation, this method can save ~18 months [4]. Although the genetic transformation system of *Eustoma* has been reported for decades, no genetically modified cultivars of *Eustoma* have been commercialized to date.

Clustered regularly interspaced short palindromic repeats-associated protein 9 (CRISPR-Cas9) technology provides a sufficient tool to target genes directly and facilitates the trait engineering of ornamental plants. Genome editing has been reported in ornamental plants such as *Phalaenopsis*, lily, chrysanthemum, petunia, carnation, and rose so far [5]. However, the stability and repeatability of these systems are questionable, since successful ornamental trait modification by genome editing has only been reported in a few species. For example, knock-out of *dihydroflavonol-4-reductase-B* blocked the synthesis of anthocyanin and led to the production of white Japanese morning glory. Knock-out of *PMADS3* and *FBP6* in petunia resulted in double-flowered phenotype [6].

Genome editing of polyploid plants with complex genetic backgrounds is more difficult. In chrysanthemum, researchers used multicopy transgenes instead of targeting endogenous genes to monitor genome editing events. For ornamental plants hard to regenerate, genome editing with protoplast becomes an alternative method to study gene function. By optimizing the CRISPR-Cas9 promoters, *EgPDS* editing was achieved in the *Eustoma* protoplasts, with editing efficiencies of 37.7% and 38.3% for the two optimized vectors [7]. However, limited by the weak regeneration and transformation system, successful genome-editing plantlet has not been reported for *Eustoma* so far.

To facilitate genetic transformation in *Eustoma*, we first established the somatic embryogenesis system for this species. Cotyledon and hypocotyl explants from seedlings of *Eustoma* were dissected and cultured on callus induction medium containing 1.5 mg/l of 2,4-dichlorophenoxyacetic acid in the dark. Plenty of golden and firm embryogenic calli (EC) were observed after ~30 days. These calli were gently fragmented and transferred on somatic embryo differentiation medium (DM) containing 0.5 mg/l of 6-benzylaminopurine (6-BA) and cultured continuously in the dark. Somatic embryos (SE) were observed after 3 weeks and the SE-derived seedlings were obtained after an additional 20-day culture under light conditions (Fig. 1a).

**Figure 1:**
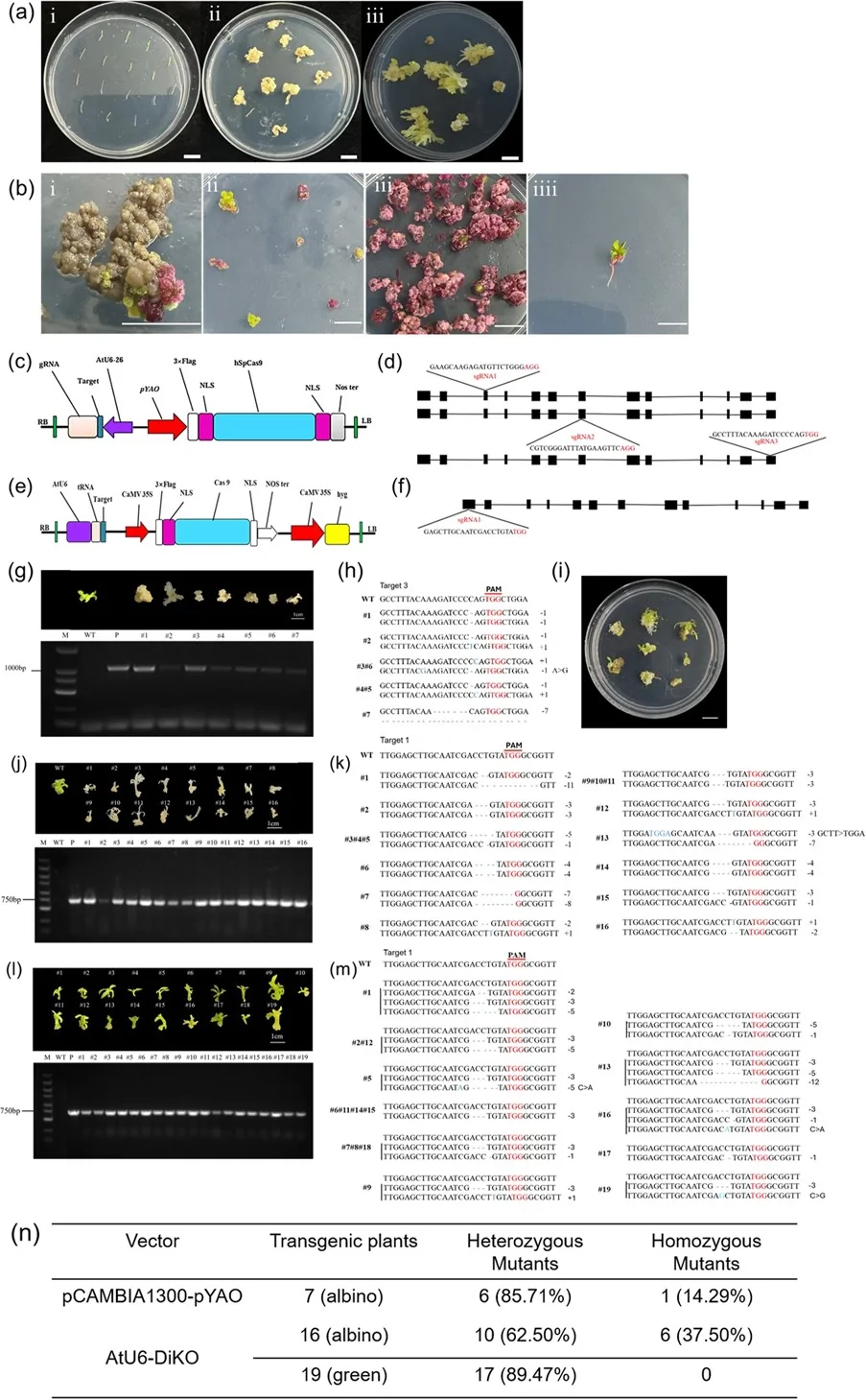
CRISPR/Cas9-mediated knockout of the *EgPDS* in *E. grandiflorum*. (a) Embryogenic callus induction of hypocotyl in *Eustoma*. i: Freshly excised hypocotyl explants prepared for callus induction; ii: callus morphology following a 30-day induction period; iii: adventitious shoots regenerated on the DM. Scale bars = 1 cm. (b) *Agrobacterium*-mediated *Eustoma* transformation was established based on the RUBY marker. i: The embryonic callus after *Agrobacterium* infection was transferred to the hygromycin screening medium for one month. ii: The surviving callus was transferred to the new proliferation screening medium. iii: Proliferation screening for 42 days of callus. iv: Regenerated plantlet cultured on rooting medium for 34 days. (c and d) Construction schematic of pCAMBIA1300-pYAO:Cas9 vector and target site screening of *EgPDS*. Scale bars = 1 cm. (e and f) Construction schematic of AtU6-Diko vector and target site screening of *EgPDS*. (g) pCAMBIA1300-pYAO:Cas9 transgenic plantlets and corresponding PCR validation. M, marker; P, plasmid used as positive control; WT, wild-type; #1–#7, Transgenic plantlets. Scale bars = 1 cm. (h) Genotype of gene-edited *EgPDS* knock out in ‘Champagne’ by pCAMBIA1300-pYAO:Cas9. Reference WT sequence was shown on top. Edited sequences (#1–#7) indicate deletions (dashes), insertions, and substitutions marked with ‘−’, ‘+’, and ‘>’ symbols, respectively. (i) Shoot regeneration from AtU6-Diko-transformed callus on hygromycin-selective medium (50 mg/l). (j–l) AtU6-Diko transgenic albino and green plantlets and corresponding PCR validation. Scale bars = 1 cm. (k–m) Genotype of gene-edited *EgPDS* knock out in ‘Light Purple’ by AtU6-Diko. Reference WT sequence was shown on top. Edited sequences of albino plantlets (#1–#16) and green plantlets (#1–#19) indicate deletions (dashes), insertions, and substitutions marked with ‘−’, ‘+’, and ‘>’ symbols, respectively. (n) Statistical analysis of *EgPDS* editing efficiency in *Eustoma*.

An *A. tumefaciens*-mediated transformation system was established based on the somatic embryogenesis and RUBY marker. We tested the effects of optical density (OD) value (0.6, 0.8, and 1.0) and EC culture duration (30, 40, and 60 days) on *Eustoma* transformation and obtained a suitable transformation system for this species. *Agrobacterium tumefaciens* suspension carrying the RUBY marker system was resuspended with Murashige and Skoog (MS) liquid containing 100 μmol/l AS. EC after about 30-day induction was cultured in preculture medium containing 50 μmol/l Acetosyringone (AS) for 3–5 days and then shaken in the resuspension (OD = 0.6) for 20 min. The infected EC were then cocultured with *A. tumefaciens* for 3–5 days and transferred to DM containing 0.5 mg/l 6-BA, 50 mg/l hygromycin (Hyg), and 400 mg/l ceftazidime. Visible red EC were isolated from the browning EC and transferred to fresh DM every 3 weeks. Hyg-resistant SEs were obtained after about 2 months (Fig. 1b).

To investigate the genetic transformation method’s feasibility for *Eustoma* genome editing, we we chose *EgPDS* with the accession number PZ236395 and designed three sgRNAs of *EgPDS* and integrated them on CRISPR-Cas9 expression vectors pCAMBIA1300-pYAO:Cas9respectively (Fig. 1c and d). *Agrobacterium tumefaciens* carrying different sgRNAs were co-transformed into EC of *Eustoma* cultivar ‘Champagne’. Seven albino and Hyg-resistant plantlets were regenerated from the EC, followed by PCR validation (Fig. 1g). We then amplified different parts of the *EgPDS* covering the three targeting sites and confirmed the sequence by Sanger sequencing. The genome editing rate of the PCR positive plantlets was 100% (one homozygote and six heterozygotes) and all the mutations occurred at the third targeting site on the 13th exon. Different mutation types were observed, including single nucleotide insertions and deletions, single nucleotide substitutions, and large fragment deletions (Fig. 1h and n, Fig. S1).

To further optimize the genome editing system, we tested the newly reported CRISPR-Cas9 vector AtU6-Diko [6]. An SgRNA targeted on Exon 1 was constructed into this vector and transformed *via A. tumefaciens* following the same protocol as above (Fig. 1e and f). Plenty of Hyg-resistant plantlets were produced on the EC displaying green or albino phenotype (Fig. 1i). We selected 35 plantlets (16 albino plantlets and 19 green plantlets) for further validation. PCR results confirmed that all the 35 plantlets were transformed (Fig. 1j and l). Further sequence validation by sanger sequencing indicated that 17 out of the 19 green plantlets were targeted and the other 2 plantlets kept WT sequence. Despite the abundant editing types on the targeting sites, a WT *EgPDS* allele was remained in the 17 green plantlets, explaining the green phenotype of those plantlets. In contrast, both *EgPDS* alleles of the 16 albino plantlets were edited with large fragment deletions. Moreover, 6 of them (37.5%) were homozygous mutations (Fig. 1i–n, Fig. S2).

In summary, this study presents an efficient gene editing system for *E. grandiflorum* based on the somatic embryogenesis system, which provides a precise genetic modification tool for molecular design breeding of *E. grandiflorum*.

## Supplementary Material

Web_Material_uhag197

## Data Availability

All the information and data supporting the results reported in this paper are available at https://pan.baidu.com/s/1BImcQ5Wj2ni76fDRr6ZqSg?pwd=xpsc. *EgPDS* had submit to GenBank with the accession number PZ236395.
